# Order Substitutions and Education for Balanced Crystalloid Solution Use in an Integrated Health Care System and Association With Major Adverse Kidney Events

**DOI:** 10.1001/jamanetworkopen.2022.10046

**Published:** 2022-05-03

**Authors:** Joseph Bledsoe, Ithan D. Peltan, R. J. Bunnell, Samuel M. Brown, Al Jephson, Danielle Groat, Nicholas M. Levin, Emily Wilson, Jon Newbold, Gabriel V. Fontaine, Joe Frandsen, David Hasleton, Paul Krakovitz, Kim Brunisholz, Todd Allen

**Affiliations:** 1 Department of Emergency Medicine, Intermountain Healthcare, Salt Lake City, Utah; 2 Department of Emergency Medicine, Stanford Medicine, Palo Alto, California; 3 Intermountain Medical Center, Salt Lake City, Utah; 4 Division of Pulmonary/Critical Care, Department of Medicine, University of Utah, Salt Lake City; 5 Department of Medicine, Intermountain Healthcare, Salt Lake City, Utah; 6 Division of Emergency Medicine, Department of Surgery, University of Utah, Salt Lake City; 7 Divisions of Epidemiology and Infectious Disease, Department of Medicine, University of Utah School of Medicine, Salt Lake City; 8 Department of Pharmacy, Intermountain Healthcare, Salt Lake City, Utah; 9 Care Transformation Information Services, Intermountain Healthcare, Salt Lake City, Utah; 10 Specialty Based Care, Intermountain Healthcare, Salt Lake City, Utah; 11 Healthcare Delivery Institute, Intermountain Healthcare, Salt Lake City, Utah; 12 Office of Quality and Patient Safety, The Queens Healthcare Systems, Honolulu, Hawaii

## Abstract

**Importance:**

Trials comparing balanced crystalloids with normal saline have yielded mixed results regarding reductions in kidney complications and mortality for hospitalized patients receiving intravenous fluids.

**Objective:**

To evaluate the association of a multifaceted implementation program encouraging the preferential use of lactated Ringer solution with patient outcomes and intravenous fluid–prescribing practices in a large, multilevel health care system.

**Design, Setting, and Participants:**

This type 2 hybrid implementation and comparative effectiveness study enrolled all patients 18 years or older who received 1 L or more of intravenous fluids while admitted to an emergency department and/or inpatient unit at 1 of 22 hospitals in Idaho and Utah between November 1, 2018, and February 29, 2020. An interrupted time series analysis was used to assess study outcomes before and after interventions to encourage use of lactated Ringer solution.

**Exposures:**

Implementation program combining order set modification, electronic order entry alerts, and sequential clinician-targeted education to encourage prescribing of lactated Ringer solution instead of normal saline.

**Main Outcomes and Measures:**

The primary implementation outcome was the patient-level proportion of intravenous fluids that was balanced crystalloids. The primary effectiveness outcome was the incidence of major adverse kidney events (MAKE30)—a composite of new persistent kidney dysfunction, new initiation of dialysis, and death—at 30 days.

**Results:**

Among 148 423 patients (median [IQR] age, 47 [30-67] years; 91 302 women [61%]), the proportion of total fluids received that was lactated Ringer solution increased from 28% to 75% in the first week vs the last week of the study (immediate implementation effect odds ratio [OR], 3.44; 95% CI, 2.79-4.24). The estimated MAKE30 absolute risk reduction was 2.2% (95% CI, 1.3%-3.3%) based on interrupted time series analysis showing a decrease in the week-on-week trend for MAKE30 (OR difference, 0.03; 95% CI, 0.03-0.03, *P* < .001). The immediate postimplementation OR for MAKE30 was 0.88 (95% CI, 0.76-1.01), with a decrease in persistent kidney dysfunction (OR, 0.80; 95% CI, 0.69-0.93) and mortality (OR, 0.78; 95% CI, 0.65-0.93) but not dialysis (OR, 1.00; 95% CI, 0.76-1.32).

**Conclusions and Relevance:**

In this comparative effectiveness study, an implementation program was associated with an increase in the proportion of fluids administered as lactated Ringer solution compared with normal saline and was associated with a reduction in MAKE30 events among patients treated in a large integrated health care system.

## Key Points

**Question:**

Is an implementation program encouraging use of balanced crystalloid infusions for fluid therapy associated with changes in type of fluids used and in the rate of major adverse kidney events at 30 days among patients treated in an emergency department or hospital setting?

**Findings:**

In this implementation and comparative effectiveness study of 148 423 patients, the proportion of total fluid received as lactated Ringer solution increased by 47% after an implementation intervention including education, order set modifications, and electronic order entry alerts. In an interrupted time series analysis, there was an observed 2.2% absolute risk reduction of major adverse kidney events after the intervention.

**Meaning:**

In this study, an implementation program in a large integrated health care system was associated with an increase in the proportion of balanced crystalloids received among patients in the emergency department and hospital settings and was associated with a reduction in major adverse kidney events.

## Teaser

This comparative effectiveness research study investigates the association of order substitutions and education for the use of lactated Ringer solution with major adverse kidney events in an integrated health care system.

## Introduction

Intravenous fluids are the most commonly prescribed medical therapy in the US, with an estimated 200 million liters of crystalloid administered annually.^1,2^ Emerging evidence suggests that normal saline (NS; 0.9% sodium chloride) solution is associated with hyperchloremic metabolic acidosis, increases in proinflammatory cytokines, decreased kidney perfusion, acute kidney injury, and mortality.^3,4,5,6,7,8,9,10^ Nevertheless, outcomes associated with different crystalloid formulations have historically been sparsely studied.

Recently, 2 randomized trials from a single center suggested preferentially administering balanced crystalloids instead of NS for almost all intravenous fluid therapy for emergency department (ED) and hospital inpatients decreases the rate of major adverse kidney events at 30 days (MAKE30), a composite outcome that comprises death, new persistent kidney dysfunction, or new dialysis within 30 days.^11,12^ Notably, these studies’ predominant balanced crystalloid was lactated Ringer (LR) solution, and study interventions began immediately on ED arrival. In contrast, the 0.9% NS vs Plasma-Lyte 148 (Baxter International) for intensive care unit (ICU) fluid therapy (SPLIT) and the Balanced Solutions in Intensive Care Study (BASICS) studies randomly assigned patients to saline solution or Plasma-Lyte after ICU admission and demonstrated no between-group differences for the primary outcomes of acute kidney injury and mortality, respectively.^13,14^

To date, the real-world effectiveness of using LR as the preferred intravenous fluid for patients throughout ED- and hospital-based care remains unknown. Furthermore, strategies for implementing a transition from preferentially prescribing NS to balanced crystalloid fluids have not, to our knowledge, been reported. We therefore performed a prospective type 2 hybrid implementation and comparative effectiveness research study—a design that gives equal weight to clinical and implementation outcomes—to evaluate practice change and clinical outcomes associated with a multimodal intervention program designed to transform intravenous fluid prescribing practices across an integrated health care system.

## Methods

### Setting and Study Population

This study was approved by the Intermountain Healthcare institutional review board with a waiver of informed consent owing to the study representing no more than minimal harm. Intermountain Healthcare is a vertically integrated health care system in Utah and Idaho with more than 550 000 ED visits annually that employs more than 10 000 nurses, 2400 physicians and advanced-practice clinicians, and another 3800 affiliated physicians and advanced-practice clinicians (eFigure 1 and eTable 1 in the Supplement). The system’s 24 hospitals include a teaching and referral hospital, 3 regional referral hospitals, 9 urban and suburban community hospitals, an orthopedic specialty hospital, 8 rural community/critical access hospitals, a pediatric specialty hospital, and a virtual telehospital, providing care for patients physically residing in one of the other hospitals or alternate care venue. The latter 2 hospitals were not included in the implementation or analysis (eTable 1 in the Supplement). Patients 18 years of age or older admitted to the ED or inpatient service on any medical/surgical unit or ICU at 1 of the 22 adult hospitals from November 1, 2018, to February 29, 2020, were included in the study upon receiving at least 1000 mL of intravenous fluids during their ED and/or inpatient stay. Patient race and ethnicity data are collected as a part of routine clinical care at the time of registration through patient self-report. In this study, race and ethnicity categories included Black, Hispanic, White, other (ie, Alaska Native, American Indian, Asian, Native Hawaiian, Pacific Islander, multiracial) and unknown. Patients admitted during the washout period from June 23 to August 1, 2019, at the time of the core implementation intervention were excluded. Only the first eligible encounter for each patient was included. Patients were categorized based on the highest level of care received during their index hospitalization. This manuscript was written according to the Standards for Reporting Implementation Studies (StaRI) guidelines.

### Study Design

We sought to simultaneously investigate the clinical outcomes associated with preferential use of balanced crystalloid for fluid resuscitation and the relative implementation association of clinician education vs electronic health record (EHR)–based strategies with balanced crystalloid adoption within a heterogeneous health care system. Therefore, we performed a prospective type 2 hybrid implementation and comparative effectiveness study with co–primary implementation (crystalloid administration practice) and clinical effectiveness outcomes. The tested interventions were designed after working with stakeholders to identify key leverage points and perceived and known barriers to the use of LR solution across the entire crystalloid administration life cycle, from crystalloid acquisition from suppliers to end-user adoption. Study interventions were designed specifically to overcome the identified barriers (eTable 2 and eFigures 2 and 3 in the Supplement).

### Intervention

To promote a transition from intravenous fluid administration of predominantly NS to predominantly LR solution, we deployed an implementation program combining clinician-focused education with modifications to our EHR order-entry system and system supply chain. EHR-based implementation took effect July 1, 2019, and comprised 2 interventions.

After review by a clinical leader designated for each specialty, standard and disease-specific EHR order sets were revised to replace bolus or maintenance NS with LR solution when there was no contraindication to therapeutic interchange. Order sets used in settings where potential drug-drug interactions were very likely (ie, in the cardiac catheterization laboratory) or related to conditions that may cause elevated intracranial pressure were not modified (eFigure 4 in the Supplement).

#### Best-Practice Alert

For NS administration orders entered outside an approved order set, we implemented a pop-up best-practice alert (eFigure 5 in the Supplement) that advised clinicians of the possible benefits of LR solution as well as its potential contraindications. The ordering clinician was required to respond with a decision to either approve therapeutic interchange to an equivalent volume and/or rate of LR solution or continue with the original order for NS. Alerts were suppressed if a patient’s serum sodium level was less than 125 mmol/dL (to convert to milliequivalents per liter, divide by 1), serum potassium was greater than 6 mmol/dL (to convert to milliequivalents per liter, divide by 1), or the active problem list suggested that the patient had or was at risk for elevated intracranial pressure.

#### Logistical Intervention

The system-level supply chain organization modified ordering and hospital-level stocking practices, including negotiating a decrease in the price of LR fluid bags to match the previously lower price of NS. At the unit level, stocking of LR solution was increased, and prepositioned stocks of NS were replaced with LR. For instance, LR solution replaced NS in bedside supply carts in EDs across our health system. NS was not replaced with LR solution in cardiac arrest supply carts owing to potential drug incompatibilities.

#### Education Intervention

All health care professionals received multifaceted education, including preimplentation messaging from hospital leadership endorsing the transition to LR solution and standard education regarding LR drug incompatibilities and contraindications. Other education was tailored to the role of the health care professional and included education on the preferred prescription of balanced crystalloids (eFigures 5, 6, and 7 in the Supplement).

In order to evaluate the importance of clinician education relative to EHR-based implementation strategies, hospitals and affiliated clinicians were assigned to receive direct education during a 3-month period either before or after EHR-based interventions based on regional patterns of shared staffing and patient referral (eFigure 2 in the Supplement). Education occurred via in-person town hall–style meetings, division or departmental meetings, and distribution of training materials via email.

ED- and hospital-based nurses completed a mandatory computer-based training during the 3 months preceding EHR-based implementation and received additional education through standard unit-based educational mechanisms. Pharmacists and affiliated personnel attended in-person or video-based lectures as part of their standard educational conferences.

### Data Sources

Data for this study were obtained from the Intermountain electronic data warehouse and validated by manual chart review by study investigators (J.B., R.J.B., I.D.P.). Mortality data were obtained via a preexisting linkage of Utah State death records and the Social Security Death Index to the electronic data warehouse. Baseline creatinine was the lowest value within the past year preceding index hospitalization. When this value was not available, baseline creatine was estimated using the Modification of Diet in Renal Disease equation.^15,16^ The Third International Consensus Definitions for Sepsis and Septic Shock (Sepsis-3) guidelines were used to identify sepsis during the encounter, using an internally validated data query (eMethods in the Supplement).^17^

### Study Outcomes

The co–primary outcomes were (1) a clinical effectiveness outcome, MAKE30, and (2) an implementation outcome, the patient-level fraction of bolus or continuous crystalloid fluid received that was a balanced crystalloid (LR or Plasma-Lyte). Secondary clinical effectiveness outcomes were the individual components of MAKE30: all-cause 30-day mortality, new persistent kidney dysfunction (defined by creatinine ≥200% of baseline at the time of the latest-available measurement, including postdischarge measurements, up to 30 days after enrollment), and new dialysis at 30 days, which included the receipt of hemodialysis or continuous kidney replacement therapies between enrollment and 30 days afterward. Patients receiving either hemodialysis or continuous kidney replacement therapies with eventual recovery of kidney function were included in this outcome. Patients with baseline end-stage kidney disease were ineligible for the new persistent kidney dysfunction and dialysis outcomes. The secondary implementation outcome was the proportion of pop-up alerts that resulted in a change in the order from NS to LR solution.

### Statistical Analysis

Patients admitted during a washout period extending from 1 week before to 1 month after the EHR intervention were excluded from analysis. Descriptive statistics, including data missingness, were calculated for demographics, baseline clinical characteristics, and fluids received during the encounter. The analysis of the association of the intervention with study outcomes employed an interrupted time series framework. The interrupted time series is a powerful nonrandomized analysis strategy that can assess the outcome of an intervention when a randomized trial is not feasible or ethical. This approach controls for secular trends over time and has a means to correct for autocorrelation if any is detected.^18,19^ LR proportion of fluid and frequency of clinical outcomes were organized in a weekly fashion to facilitate the interrupted time series analyses.

Before interrupted time series models were created, we first risk-adjusted clinical outcomes using patient characteristics selected a priori: age, sex, race and ethnicity, Charlson Comorbidity Index (calculated without kidney failure component),^20^ end-stage kidney disease (obtained from *International Statistical Classification of Diseases, Tenth Revision, Clinical Modification *[*ICD-10-CM*] codes), initial creatinine, and acute physiology score^21,22^ within the first 24 hours of hospital arrival. Risk-adjusted models were then calibrated to estimate MAKE30 at the same rate that was observed during the study. The weekly risk-adjusted outcome rate was calculated using indirect standardization. Segmented β regression was used to assess the association of education and EHR implementation with LR proportion of fluid. The model was parameterized to evaluate the association of education (which occurred for some hospital sites before EHR implementation and the remaining hospital sites afterward) and EHR implementation. The change in slope for education periods and the immediate step-off of the EHR implementation were captured with the model. Segmented fractional logistic binomial regression was used to assess the associaiton of the EHR implementation with clinical outcomes. The models were parameterized to capture the trend of the incidence rate before and after, as well as the immediate outcome of the EHR changes (eMethods in the Supplement). Autocorrelation was assessed with the Durbin-Watson statistic and accounted for if necessary. Additional details are provided in eMethods in the Supplement.

The end-of-study incidence rates for the MAKE30 outcome and each of its components estimated from post-EHR implementation data were compared with the counterfactual incidence rate at the same time point projected from the preimplementation trends, ie, as if the intervention had not occurred. Estimated incidence rates were obtained via 10 000 bootstrap iterations by selecting random β values from the multivariate normal distribution based on the covariance matrix from the interrupted time series models. The incidence rate at the end of the study and the counterfactual calculated from each model over the bootstrap iterations provided point estimates for the absolute and relative outcome differences along with the associated 95% CIs. Interrupted time series analysis and predifferences vs postdifferences were also repeated after stratification across prespecified subgroups including admission type (ICU, ED, inpatient surgery, inpatient), age, sex, hospital affinity (tertiary, community, rural), total fluid volume, sepsis, and traumatic brain injury.

Monte Carlo simulations (10 000 iterations) with a population of 118 000 demonstrated 80% power to detect an absolute reduction greater than 0.4% for clinical outcomes when considering a baseline incidence rate of 3.8%. A 2-sided *P* value ≤ .05 was considered significant. Analyses were performed with R, version 4.0.3 (R Foundation).

## Results

After excluding patients arriving to a hospital during the washout period, included in the study were a total of 148 423 adult patients (median [IQR] age, 47 [30-67] years; 91 302 women [61%]; 57 121 men [39%]) who were admitted to a study ED or hospital between November 1, 2018, and February 29, 2020, and received 1000 mL or more of intravenous crystalloid throughout the entirety of their encounter. Patients of the following race and ethnicity categories were included: 1935 Black (1%), 2899 Hispanic (2%), 134 623 White (91%), 5866 patients (4%) in the other category and 3100 patients (2%) with unknown race and ethnicity. Patients were similar in the preimplementation and postimplementation cohorts with regard to median (IQR) age (47 [31-67] years vs 46 [30-66] years), female sex (53 608 [62.1%] vs 37 694 [60.8%]), median (IQR) acute physiology score on presentation (4 [2-6] vs 4 [2-6]), median (IQR) Charlson Comorbidity Index score (1 [0-3] points vs 1 [0-2] points) as well as race and ethnicity and encounter type (Table 1). Frequency of *ICD-10-CM* discharge diagnosis codes is reported in eTable 3 in the Supplement.

**Table 1.** Demographics, Baseline Characteristics, and Unadjusted Outcomes by Implementation Phase

Characteristic	No. (%)	
	Preimplementation (n = 86 388)	Postimplementation (n = 62 035)
Age, median (IQR), y	47 (31-67)	46 (30-66)
Sex		
Female	53 608 (62.1)	37 694 (60.8)
Male	32 780 (37.9)	24 341 (39.2)
Race and ethnicity		
Black	1105 (1.3)	830 (1.3)
Hispanic	1502 (1.7)	1397 (2.3)
White	78 943 (91.4)	55 680 (89.8)
Other^a^	3368 (3.9)	2498 (4)
Unknown	1470 (1.7)	1630 (2.6)
Encounter type		
ED only	46 115 (53.4)	33 642 (54.2)
ICU inpatient	5662 (6.6)	3709 (6)
Medicine inpatient	22 619 (26.2)	16 036 (25.8)
Surgery inpatient	11 992 (13.9)	8648 (13.9)
Hospital affinity		
Tertiary	48 798 (56.5)	34 354 (55.4)
Community	29 279 (33.9)	21 195 (34.2)
Rural	8311 (9.6)	6486 (10.5)
Serum creatinine, median (IQR), mg/dL^b^	0.8 (0.6-0.9)	0.8 (0.6-0.9)
Serum creatinine calculated	41 976 (48.6)	36 758 (59.3)
Acute physiology score, median (IQR)	4 (2-6)	4 (2-6)
Charlson comorbidity index, median (IQR), points	1 (0-3)	1 (0-2)
Chronic kidney disease	10 795 (12.5)	6429 (10.4)
End-stage kidney disease with dialysis	267 (0.3)	41 (0.1)
Traumatic brain injury	810 (0.9)	733 (1.2)
Sepsis	5086 (5.9)	3356 (5.4)
Total fluid volume		
Median (IQR), mL	1030 (1000-2197)	1020 (1000-2040)
≥2000 mL	28 776 (33.3)	19 474 (31.4)
Normal saline volume		
Median (IQR), mL	1000 (600-1469)	20 (0-381)
≥2000 mL	18 149 (21)	4502 (7.3)
Balanced crystalloid volume		
Median (IQR), mL	0 (0-1000)	1000 (1000-1800)
≥2000 mL	9841 (11.4)	14 396 (23.2)
% Plasma-Lyte 148, median (IQR)	5.64 (3.56-7.4)	1.17 (0.96-1.4)
Outcomes		
Fluid volume consistent with study phase, %		
100	58 160 (67.3)	29 083 (46.9)
51-99	7566 (8.8)	21 394 (34.5)
1-50	13 272 (15.4)	3750 (6)
0	7390 (8.6)	7808 (12.6)
Major adverse kidney event at 30 d	2469 (2.9)	1495 (2.4)
New persistent kidney dysfunction	2174 (2.5)	1323 (2.1)
New dialysis	574 (0.7)	376 (0.6)
All-cause mortality	1606 (1.9)	1028 (1.7)

Abbreviations: ED, emergency department; ICU, intensive care unit.

^a^ Other category includes Alaska Native, American Indian, Asian, Native Hawaiian, Pacific Islander, and multiracial.

^b^ To convert to micromoles per liter, multiply by 88.4.

**Figure 1.**
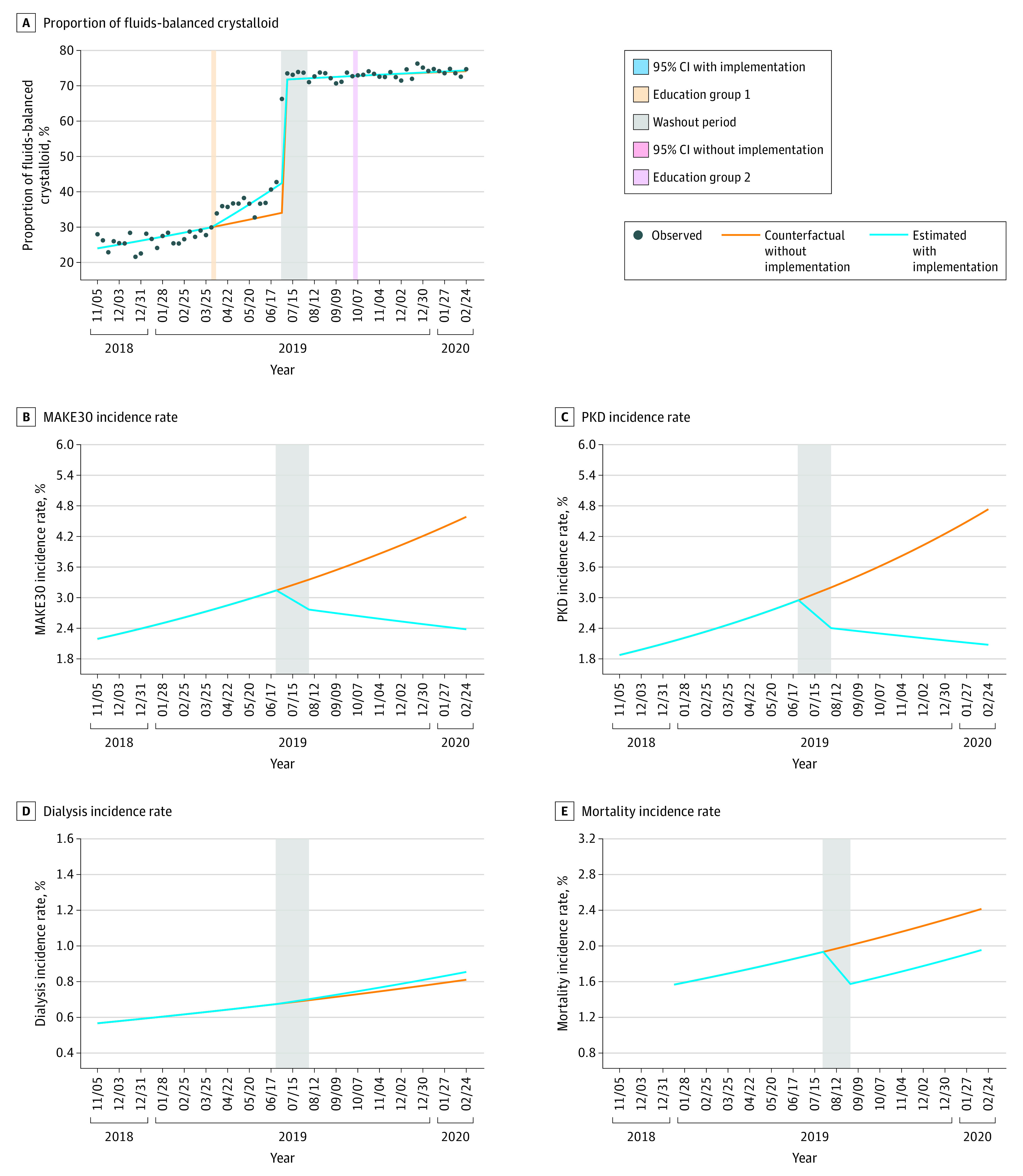
Interrupted Time Series Estimated Actual and Counterfactual Incidence Rates A, Proportion of fluids-balanced crystalloids with and without education implementation; B, major adverse kidney events at 30 days (MAKE30) incidence rate; C, persistent kidney dysfunction (PKD) incidence rate; D, dialysis incidence rate; and E, mortality incidence rate.

The median (IQR) volume of NS infused in the preimplementation period was 1000 (600-1469) mL; 65 726 of 86 388 (76.1%) patients received more than half of their total fluids as NS. Postimplementation, the median (IQR) balanced crystalloids volume infused was 1000 (1000-1800) mL, and 50 477 of 62 035 (81.4%) patients received more than half of their total fluids as balanced crystalloids (Table 1). The interrupted time series analysis demonstrated a week-on-week increase in the proportion of intravenous fluid administered that was balanced crystalloid that accelerated after clinician education began at sites where education occurred before the EHR implementation (Table 2 and Figure 1). There was an increase in the proportion of fluid received as balanced crystalloids associated with EHR implementation (odds ratio [OR], 3.44; 95% CI, 2.79-4.24). Balanced crystalloids administration proportion was stable after EHR implementation. Over the course of the study, the observed proportion of all fluids received that were balanced crystalloid increased from 28% during the first week to 75% during the last week (Figure 1). After the EHR implementation, 11.9% of NS orders (633 of 5316) occurring outside an approved order set were changed after the best-practice alert fired.

**Table 2.** Weekly Change, Immediate Change, Risk-Adjusted, and Counterfactual Estimates for Clinical and Implementation Outcomes

Outcome	Odds ratio (95% CI)	Estimate, % (95% CI)	*P* value
**Weekly incidence trend pre–EMR implementation**			
Major adverse kidney event	1.01 (1.01 to 1.02)	NA	<.001
Persistent kidney dysfunction	1.01 (1.01 to 1.02)		<.001
Dialysis	1.01 (1.00 to 1.01)		.23
All-cause mortality	1.01 (1.00 to 1.01)		.01
**Immediate change with EMR implementation**			
Major adverse kidney event	0.88 (0.76 to 1.01)	NA	.06
Persistent kidney dysfunction	0.80 (0.69 to 0.93)		.004
Dialysis	1.00 (0.76 to 1.32)		.99
All-cause mortality	0.78 (0.65 to 0.93)		.005
**Weekly incidence trend post–EMR implementation**			
Major adverse kidney event	0.98 (0.98 to 0.99)	NA	<.001
Persistent kidney dysfunction	0.98 (0.97 to 0.99)		<.001
Dialysis	1.00 (0.99 to 1.02)		.83
All-cause mortality	1.00 (0.99 to 1.01)		.80
**Estimated end-of-study incidence with EMR implementation**			
Major adverse kidney event	NA	2.38 (2.15 to 2.63)	NA
Persistent kidney dysfunction		2.08 (1.86 to 2.31)	
Dialysis		0.85 (0.72 to 1.02)	
All-cause mortality		1.95 (1.74 to 2.19)	
**Estimated end-of-study incidence without EMR implementation**			
Major adverse kidney event	NA	4.58 (3.72 to 5.63)	NA
Persistent kidney dysfunction		4.73 (3.81 to 5.87)	
Dialysis		0.81 (0.52 to 1.27)	
All-cause mortality		2.41 (1.86 to 3.13)	
**Estimated end-of-study absolute difference pre– vs post–EMR implementation**			
Major adverse kidney event	NA	−2.23 (−3.29 to −1.29)	<.001
Persistent kidney dysfunction		−2.68 (−3.84 to −1.68)	<.001
Dialysis		0.03 (−0.44 to 0.39)	.91
All-cause mortality		−0.48 (−1.22 to 0.16)	.17
**Estimated end-of-study relative difference pre– vs post–EMR implementation**			
Major adverse kidney event	NA	−47.70 (−58.66 to −34.37)	<.001
Persistent kidney dysfunction		−55.78 (−65.50 to −43.91)	<.001
Dialysis		8.66 (−34.81 to 73.26)	.77
All-cause mortality		−18.18 (−39.14 to 8.24)	.13
**Balanced crystalloid proportion of fluid**			
Pre**–**EMR implementation and groups 1 and 2 pre-education trend	1.02 (1.01 to 1.02)	NA	<.001
Pre**–**EMR implementation and group 1 posteducation trend	1.03 (1.01 to 1.04)		<.001
Immediate change with EMR implementation	3.44 (2.79 to 4.24)		<.001
Post-EMR implementation and group 2 pre-education trend	0.96 (0.94 to 0.98)		<.001
Post**–**EMR implementation and groups 1 and 2 posteducation trend	1.00 (0.98 to 1.02)		.99
**Balanced crystalloid proportion of fluid: end of pre–EMR implementation**			
Estimated incidence with education	NA	41.44 (39.42 to 43.48)	NA
Estimated incidence without education		33.72 (31.05 to 36.35)	NA
Estimated absolute difference pre vs post education		7.72 (3.92 to 11.53)	<.001
Estimated relative difference pre vs post education		23.12 (10.91 to 36.73)	<.001
**Balanced crystalloid proportion of fluid: end of post–EMR implementation**			
Estimated incidence with education	NA	74.35 (72.92 to 75.78)	NA
Estimated incidence without education		74.13 (65.45 to 81.50)	NA
Estimated absolute difference pre vs post education		0.22 (−7.81 to 9.46)	.96
Estimated relative difference pre vs post education		0.62 (−8.06 to 11.43)	.94

Abbreviation: NA, not applicable.

**Figure 2.**
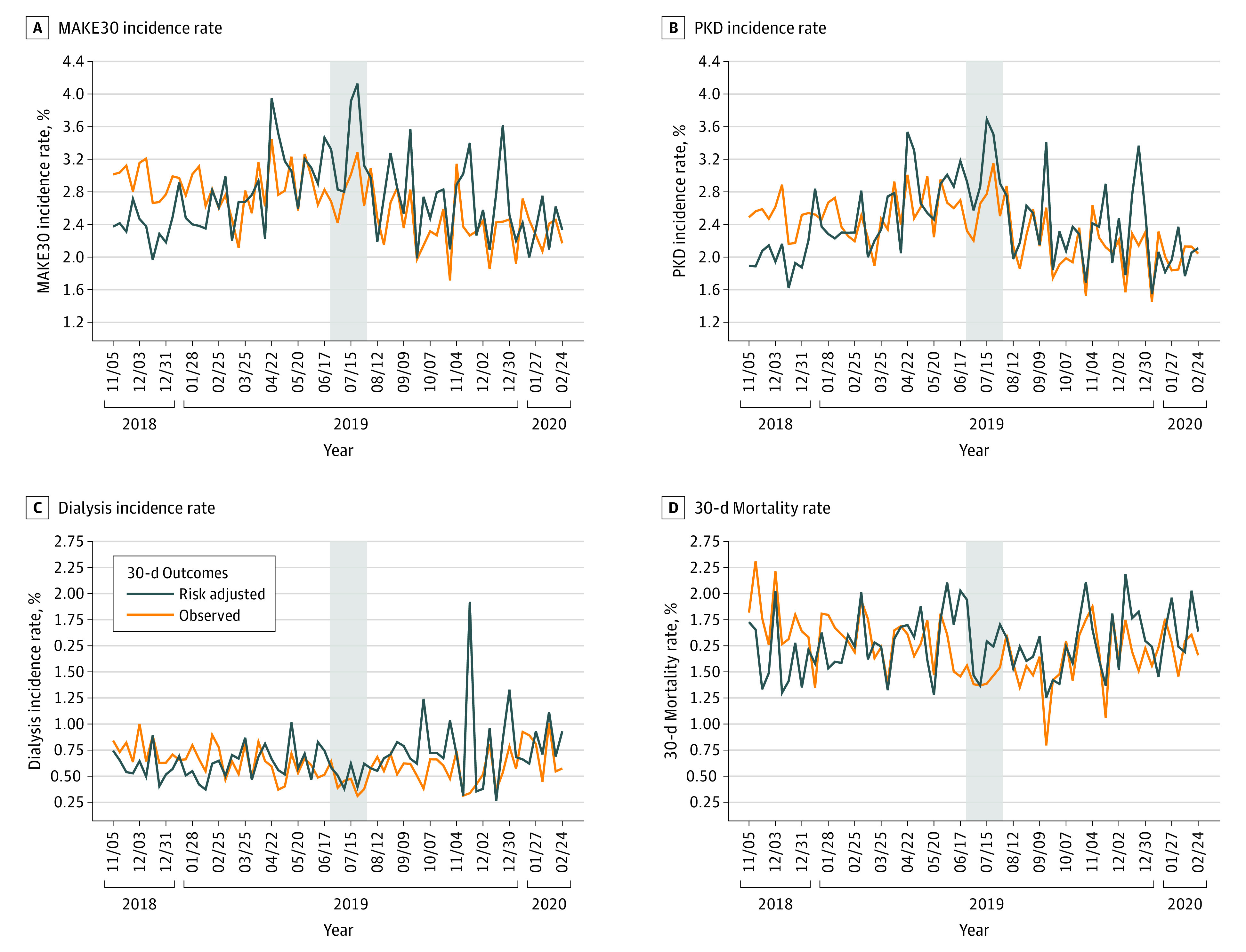
Unadjusted and Risk-Adjusted Incidence Rates for 30-Day Clinical Outcomes A, Major adverse kidney events at 30 days (MAKE30) incidence rate; B, persistent kidney dysfunction (PKD) incidence rate; C, dialysis incidence rate; and D, 30-day all-cause mortality.

**Figure 3.**
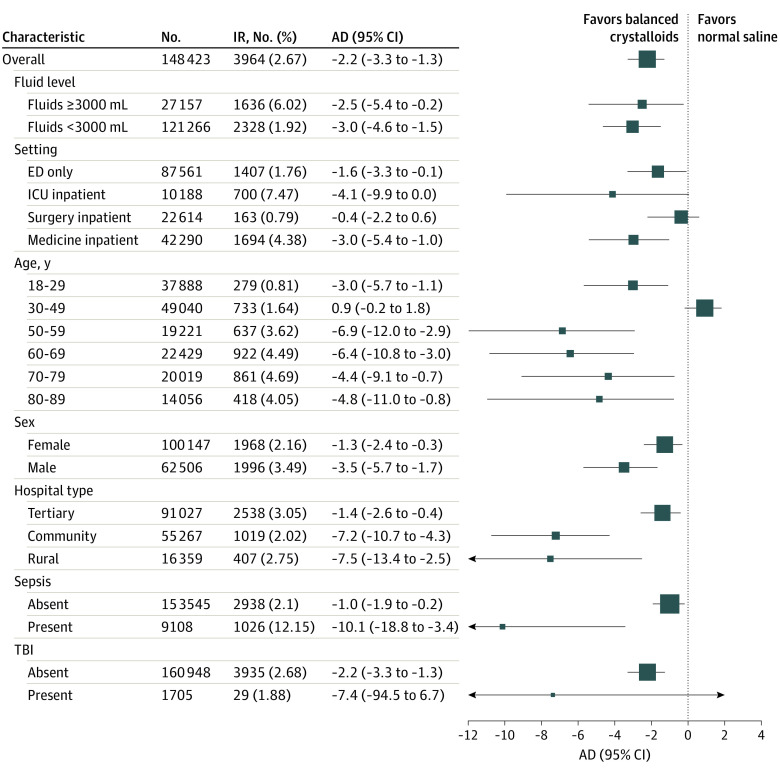
Forest Plot of Absolute Difference (AD) in Risk-Adjusted Incidence Rate (IR) of Major Adverse Kidney Events at 30 Days for Subgroups ED indicates emergency department; ICU, intensive care unit; TBI, traumatic brain injury.

In the preimplementation phase, 2469 of 86 388 patients (2.9%) had a MAKE30 event compared with 1495 of 62 035 (2.4%) in the postimplementation phase. The unadjusted incidence of MAKE30 was 2.9% (86 of 2853) during the first week of the study and 2.1% (34 of 1567) during the last week of the study. The adjusted rate of MAKE30 was 2.3% (95% CI, 1.82%-2.97%) during the first week of the study and 2.33% (95% CI, 1.61%-3.16%) during the last week of the study (Figure 2). In the interrupted time series analysis, the risk-adjusted week-on-week trend for MAKE30 decreased after the EHR order set implementation from adjusted OR of 1.01 (95% CI, 1.01-1.10) to an OR of 0.98 (95% CI, 0.98-0.99) (Table 2). The estimated MAKE30 absolute risk reduction was 2.2% (95% CI, 1.3%-3.3%) showing a decrease in the week-on-week trend for MAKE30 with an OR difference of 0.03 (95% CI, 0.03-0.03; *P* value <.001) (Figure 3). There was a significant decrease in the week-on-week incidence trend for persistent kidney dysfunction (OR, 1.01; 95% CI, 1.01-1.02 to OR, 0.98; 95% CI, 0.97-0.99). Based on weekly trends derived in the preimplementation period, there was a 47% relative reduction in estimated end-of-study MAKE30 events.

In the immediate change analysis after EHR implementation, there was an immediate (step-off) change in MAKE30 with a resultant odds ratio of 0.88 (95% CI, 0.76-1.01). In addition, there were step-off changes after EHR implementation for new persistent kidney dysfunction (OR, 0.80; 95% CI, 0.69-0.93) and mortality (OR, 0.78; 95% CI, 0.65-0.93), but not new dialysis (OR, 1.00; 95% CI, 0.76-1.32) (Table 2).

In the counterfactual analysis when comparing the end-of-study MAKE30 incidence estimated from the post–EHR implementation trend with the counterfactual incidence estimated from preimplementation trends (Figure 1), there was a reduction of MAKE30 incidence from 4.58% to 2.38% (absolute difference, −2.23%; 95% CI, −3.29 to −1.29). This yielded a number needed to treat to prevent one additional MAKE30 event of 49. The incidence rate of new persistent kidney dysfunction also decreased significantly compared with the counterfactual (absolute difference, −2.68%; 95% CI, −3.84 to −1.68), but new dialysis and 30-day all-cause mortality were unchanged. When evaluated in patient subgroups, the absolute difference in reduction of estimated end-of-study MAKE30 incidence was larger for patients with total intravenous fluid volume of 3000 mL or greater (eFigure 8 in the Supplement), sepsis vs without sepsis (eFigure 9 in the Supplement), and for ED and medical service inpatients compared with nonsignificant change in ICU and inpatient surgery patients (eFigure 10 in the Supplement) as well as age by decade (eFigure 11 in the Supplement).

## Discussion

In this large, type 2 hybrid implementation and comparative effectiveness study, we observed an increase in the use of balanced crystalloid associated with an implementation program combining clinician education with introduction via EHR of revised order sets and real-time best practice alerts. The outcome of clinician education was modest compared with EHR-based interventions. Clinical outcomes improved after implementation, with a 47% relative reduction in estimated end-of-study MAKE30 incidence, based on weekly trends derived in the preimplentation period. To our knowledge, this was the first prospective implementation study to evaluate process and clinical outcomes associated with large-scale implementation encouraging preferential prescribing of LR solution rather than NS.

Considering that fluid resuscitation is an overwhelmingly common therapy prescribed to patients in the ED and hospital inpatients and the diverse options available when administering this intervention, relatively little evidence was available until recently to guide the choice of crystalloid fluids.^23^ Our findings provide data consistent with the clinical benefit observed in recent randomized clinical trials that used predominantly LR solution and initiated the fluid choice intervention in the ED and also demonstrated the feasibility of implementation across diverse clinical settings and patient profiles. Although the absolute effect size is moderately small, with a number needed to treat of 49, the population-level outcome is very large given the large number of patients who receive crystalloid fluid therapy. The moderate patient-level effect size may explain why prior trials of balanced crystalloids vs NS that enrolled fewer patients have been negative.^14,24,25^ Our effect sizes, however, were similar to 2 recent randomized trials that applied innovative methods to achieve adequate power despite a single-center design.^11,12^

Although preferential use of balanced crystalloids appeared to exert clinical benefit across all ED and hospital adult patients in the primary analysis, results from our subgroup analyses may help institutions focus implementation efforts where broader implementation is not pursued. Relative and absolute risk reductions were particularly large in patients with sepsis, concordant with results from the subgroup analysis from the Isotonic Solutions and Major Adverse Renal Events Trial (SMART).^26^ Among patients who received higher volume infusions (>3000 mL) during their hospitalization, we observed a trend toward lower relative MAKE30 events compared with patients who received smaller volume infusions (eFigure 8 in the Supplement). Medical inpatients and ED patients not admitted to the hospital appeared to derive greater benefits than surgery patients, similar to other prior studies.^14,26,27,28,29^ Inclusion of a high proportion of elective surgical patients in the SPLIT and BaSICS trials^13,14^ may have contributed to their negative findings.^30^

Intervention timing in our study, as with previous trials, appeared to be critical for patient level benefit. Our study, the Saline Against Lactated Ringer or Plasma-Lyte in the Emergency Department (SALT-ED) study, and SMART trial initiated preferred prescription of balanced crystalloid on hospital arrival. By contrast, SPLIT and BaSICS enrolled patients only after ICU admission and similar to our study, did not find a significant outcome difference in intravenous fluids in this population. As a result of the delayed initiation, patients often received substantial off-protocol intravenous fluids before enrollment; half of the participants in BaSICS, eg, received greater than 1 L of intravenous fluid prerandomization. Taken together with the negative finding from these “late” intervention trials as well as a secondary analysis of the SMART trial that found the beneficial effect of balanced crystalloid depended on its initiation in the ED,^27^ our results suggest that early intervention may be necessary to minimize normal saline exposure and maximize clinical benefits from the preferred prescription of balanced crystalloids. The fact that patients who received balanced crystalloid in the positive trials predominantly received LR solution whereas patients in the negative trials received Plasma-Lyte 148 raises the possibility that beneficial outcomes may be associated with the specific balanced crystalloid fluid administered.

EHR-based interventions to change default intravenous fluid selection combined with logistical and supply chain interventions to increase availability of LR were associated with a large and sustained change in the proportion of balanced fluid prescribed. In contrast, clinician education delivered before these interventions was associated with only a minimal change in prescription habits, and education after these interventions exerted no discernable associations with balanced crystalloid uptake. Together with the fact that relatively few EHR “best-practice” alerts triggered ordered modification, these findings suggest that intensive education programs may be less important than pragmatic interventions to align preset defaults with and minimize the obstacles for recommended behaviors.^31,32^

For a host of reasons, medicine has had difficulty implementing new medical evidence into routine clinical practice.^33^ Although the prior prospective trials of balanced crystalloids employed strict protocols or were performed in single hospital settings, our study describes implementation across an integrated health care system, including academic institutions, referral centers, and community and rural critical access hospitals. The diverse ED and inpatient settings contribute to making our results more generalizable. The finding that an EHR-based intervention focused on modifying default orders was sufficient to substantially change crystalloid administration practices suggests the possibility of creating a scalable and potentially sustainable implementation strategy. We hope that implementation studies like this may help accelerate the capability of clinicians and systems to embed best practice in routine medical work.^34^

### Limitations

Although the timing and magnitude of the observed changes in balanced crystalloid administration make it unlikely that factors aside from the studied intervention explain the observed changes in balanced crystalloid administration, it is possible that factors other than the studied intervention may have contributed to observed changes in our clinical outcomes. We did not evaluate the association of study phase with patients lactate trajectory or with evolving sodium and chloride levels during their hospitalization. This physiologic separation of groups has been performed previously, and we did not believe our populations would differ significantly.^11^ Our risk adjustment model appeared to provide less stable outcome estimates in the ED-only subgroup. This may reflect the inherent heterogeneity of ED patients or less available postdisposition data affecting persistent kidney dysfunction estimates. Although the risk-adjustment model performed well in the overall population, the ED-only subgroup may warrant further evaluation to determine if it can be further stratified to identify patients that may be more likely to benefit from balanced crystalloids. We were not able to incorporate postdischarge creatinine measurements or dialysis initiations occurring outside our health system in outcome measurements. The study was performed in a single integrated health care system; differences in workplace culture and other system factors may alter the effectiveness of the studied implementation strategy in other settings. Although a diverse spectrum of hospital type and specialties and the associated variation in baseline care delivery support generalizability, it remains possible that practices in the studied hospitals may not be representative of those outside of our health care system. Significant clinical outcome improvements observed despite relatively high use of LR solution at baseline and incomplete adoption (compared with recent trials) of balanced crystalloids after implementation support generalizability as well. The ideal system-level target for balanced crystalloid proportion remains unclear and deserves investigation in future studies.

## Conclusions

In this type 2 hybrid implementation and comparative effectiveness study involving a 24-hospital integrated health care system, results suggest that an implementation program combining health care professional education with EHR-based alerts and order set modification was associated with an increase in the proportion of balanced crystalloids prescribed and a reduction in MAKE30 events. Although education-based interventions were associated with modest increases in the proportion of prescribed balanced crystalloids, the EHR-based interventions were associated with large increases even when these changes preceded clinician education.

## Notes

Supplement. **eFigure 1.** Map of Study Sites **eTable 1.** Study Hospital Characteristics **eTable 2.** Implementation Framework and Strategy **eFigure 2.** Flow Diagram of Study Implementation **eFigure 3.** Implementation Team Organizational Chart **eFigure 4.** Example of Electronic Health Record Order Set **eFigure 5.** Best Practice Alert **eFigure 6.** Sample Educational Materials Used for Implementation (Presentation) **eFigure 7.** Sample Educational Materials Used for Implementation (Informational Flyer)
eMethods.
**eTable 3.** ICD-10 Root Codes Occurring in 5% or More of the Study Population as the Primary (First) Discharge Diagnosis **eFigure 8.** Forest Plot of Relative Difference in Incidence Rate of MAKE30 for Subgroups **eFigure 9.** Observed and Risk-Adjusted Rate of MAKE30 in Patient Subgroups **eFigure 10.** Observed and Risk-Adjusted Incidence Rate of MAKE30 by Patient Admission Type **eFigure 11.** Observed and Risk-Adjusted Incidence Rate of MAKE30 in Patient Subgroups by Age
