# Supplementary material for: Order Substitutions and Education for Balanced Crystalloid Solution Use in an Integrated Health Care System and Association With Major Adverse Kidney Events
Source: JAMA Netw Open. 2022 May 3;5(5):e2210046. doi: 10.1001/jamanetworkopen.2022.10046 (PMC9066288; doi:10.1001/jamanetworkopen.2022.10046)
Supplement: Supplement. — eFigure 1. Map of Study Sites eTable 1. Study Hospital Characteristics eTable 2. Implementation Framework and Strategy eFigure 2. Flow Diagram of Study Implementation eFigure 3. Implementation Team Organizational Chart eFigure 4. Example of Electronic Health Record Order Set eFigure 5. Best Practice Alert eFigure 6. Sample Educational Materials Used for Implementation (Presentation) eFigure 7. Sample Educational Materials Used for Implementation (Informational Flyer) eMethods. eTable 3. ICD-10 Root Codes Occurring in 5% or More of the Study Population as the Primary (First) Discharge Diagnosis eFigure 8. Forest Plot of Relative Difference in Incidence Rate of MAKE30 for Subgroups eFigure 9. Observed and Risk-Adjusted Rate of MAKE30 in Patient Subgroups eFigure 10. Observed and Risk-Adjusted Incidence Rate of MAKE30 by Patient Admission Type eFigure 11. Observed and Risk-Adjusted Incidence Rate of MAKE30 in Patient Subgroups by Age [file jamanetwopen-e2210046-s001.pdf]

Supplementary Online Content

Bledsoe J, Peltan ID, Bunnell RJ, et al. Order substitutions and education for balanced crystalloid solution use in an integrated health care system and association with major adverse kidney events. *JAMA Netw Open*. 2022;5(5):e2210046. doi:10.1001/jamanetworkopen.2022.10046

eFigure 1. Map of Study Sites

eTable 1. Study Hospital Characteristics

eTable 2. Implementation Framework and Strategy

eFigure 2. Flow Diagram of Study Implementation

eFigure 3. Implementation Team Organizational Chart

eFigure 4. Example of Electronic Health Record Order Set

eFigure 5. Best Practice Alert

eFigure 6. Sample Educational Materials Used for Implementation (Presentation)

eFigure 7. Sample Educational Materials Used for Implementation (Informational Flyer)

eMethods.

eTable 3. ICD-10 Root Codes Occurring in 5% or More of the Study Population as the Primary (First) Discharge Diagnosis

eFigure 8. Forest Plot of Relative Difference in Incidence Rate of MAKE30 for Subgroups

eFigure 9. Observed and Risk-Adjusted Rate of MAKE30 in Patient Subgroups

eFigure 10. Observed and Risk-Adjusted Incidence Rate of MAKE30 by Patient Admission Type

eFigure 11. Observed and Risk-Adjusted Incidence Rate of MAKE30 in Patient Subgroups by Age

This supplementary material has been provided by the authors to give readers additional information about their work.

© 2022 Bledsoe J et al. *JAMA Network Open.*

eFigure 1. Map of Study Sites

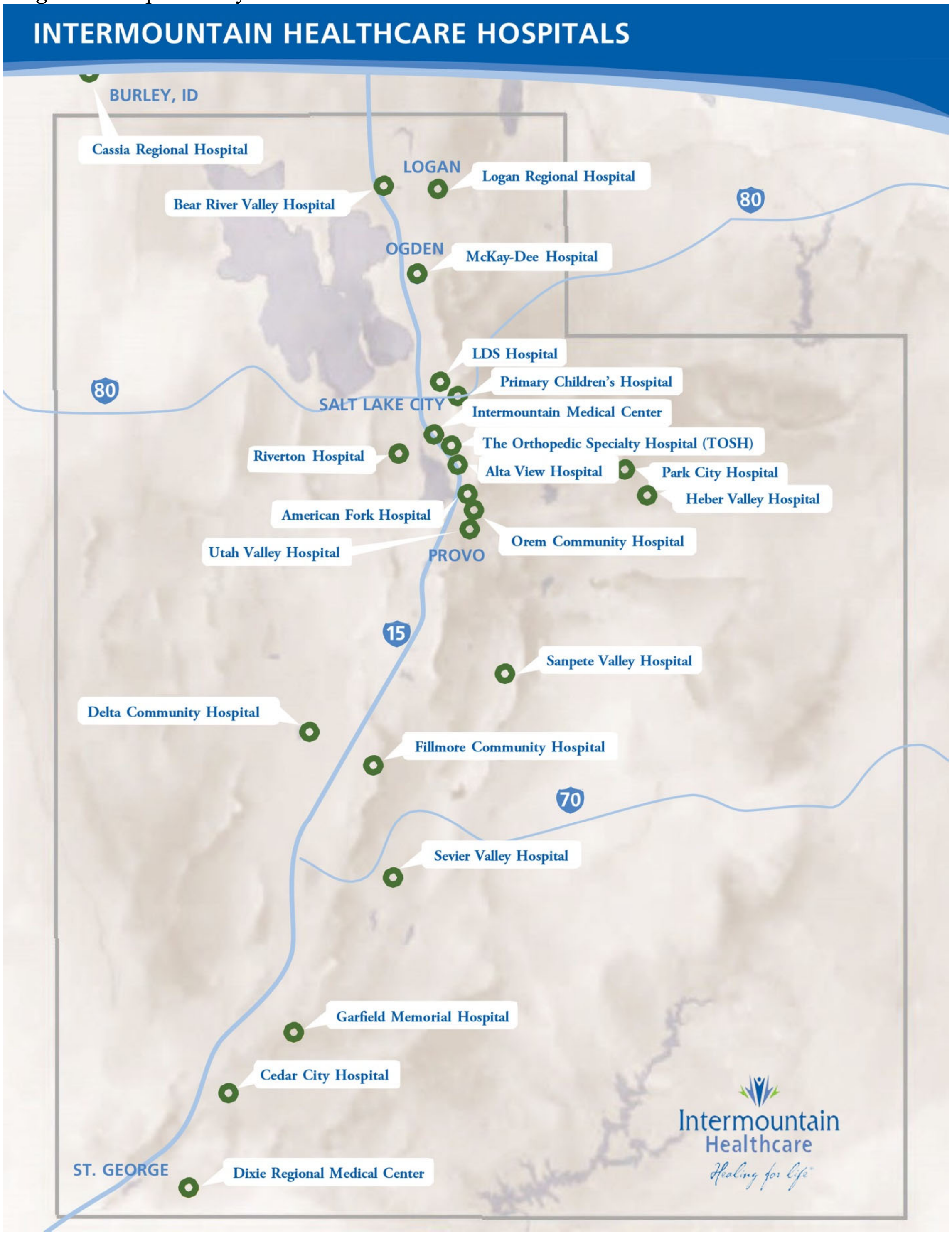

Layton Hospital (participating site) is located between Salt Lake City and Ogden, UT and is not depicted on this map. Primary Children's Hospital was excluded from study participation. St. George Medical Center (participating site) changed titles during the study period so is depicted as Dixie Regional Medical.

© 2022 Bledsoe J et al. *JAMA Network Open.*

eTable 1. Study Hospital Characteristics

Hospital	City	Beds	Trauma Level Designation	Annual ED Visits	Hospital Type	Education Phase
Intermountain Medical Center	Murray	472	TI	95,000	Tertiary	1
Utah Valley	Provo	395	TII	49,000	Tertiary	2
McKay-Dee	Ogden	321	TII	64,000	Tertiary	2
St. George Medical Center	St. George	245	TII	52,800	Tertiary	1
The Orthopedic Specialty Hospital	Murray	36		N/A	Community	1
LDS Hospital	Salt Lake City	250		24,000	Community	1
Logan Regional	Logan	146	TIII	26,400	Community	2
Riverton	Riverton	97	TIII	25,000	Community	1
American Fork	American Fork	89	TIV	31,000	Community	2
Alta View	Sandy	71	TIII	21,000	Community	1
Cedar City	Cedar City	48		19,000	Community	1
Park City	Park City	37	TIV	11,700	Community	2
Sevier Valley	Richfield	29		7,600	Rural	1
Cassia Regional	Burley, ID	25		11,000	Rural	2
Orem Community	Orem	24		7,000	Rural	2
Fillmore Community	Fillmore	19		1,700	Rural	1
Heber Valley	Heber	19		7,200	Rural	2
Delta Community	Delta	18		2,500	Rural	1
Sanpete Valley	Mt. Pleasant	18		5,200	Rural	1
Bear River Valley	Tremonton	16	TIV	6,200	Rural	2
Garfield Memorial	Panguitch	14		2,600	Rural	1
Layton	Layton	50	TIV	5,000	Rural	2

© 2022 Bledsoe J et al. *JAMA Network Open.*

eTable 2. Implementation Framework and Strategy

Implementation steps	Descriptions	Examples	Adaptations
Identify key clinical leverage points	The team identified key places to intervene in the clinical workflow to drive improvement.	Knowledge/Acceptance: Physician/APP/Nurse/Pharmacist education<br>Workflow: EHR IVF orders	Pop up alert
Identify actors and performance objectives	Actors were identified in the clinical workflow process with assigned performance objectives to support adherence to the clinical leverage points.	Clinical leaders- Letter of support<br>Clinical champion- review orders for targeted substitutions<br>Supply chain- order/stock LR to support anticipated increase in demand	Front line clinician champions identified to address slow adopters
Select from known implementation strategies and link to barriers	The team designed interventions to influence previously identified barriers to prescribing LR. This specific step allowed the team to logically follow previous steps, identifying interventions that will be more effective for influencing implementation and producing a final logic model depicting relationships.	Scale up strategy- provide access to LR (supply chain/stock bedside carts etc.)<br>Implementation process strategy- Engage key stakeholders/clinical champions<br>Dissemination strategy- evidence based intervention (EBI) distributed to clinicians	Interventions were adapted to improve acceptance
Produce protocol and implementation materials	The team created design documents, draft content, pretest and refine content, and produce final materials to achieve action targets. This design document not only supports the development of implementation plan but can also help evaluation and potential adaptation of future interventions.	Action Targets: Proportion of fluids delivered -LR<br>Incidence of MAKE-30<br>Evidence-based intervention (EBI) materials created tailored to clinical discipline<br>Expert reviewed targeted order substitutions	Final materials were tailored to specific disciplines.
Implement across system	We developed the mechanisms for which we expected the implementation strategies to work and allowed us to determine future relationships. This included working within the constraints of our EHR rules and development processes.	Phase1: Hospital group 1 education (all hospitals nurse/pharmacy education)<br>Phase 2: All hospitals EHR order substitutions/alert<br>Phase 3: Hospital group 2 education	Sustain effects with EHR-embedded solutions.
Evaluate	The team developed mechanisms for monitoring the implementation outcome. Performance feedback provided to ordering clinicians performing below the target 75% LR proportion of fluids prescribed.	A dashboard to monitor the implementation outcome using the lead measure: IV fluid prescribing.	Email to clinicians falling below target of 75%.

© 2022 Bledsoe J et al. *JAMA Network Open.*

eFigure 2. Flow Diagram of Study Implementation

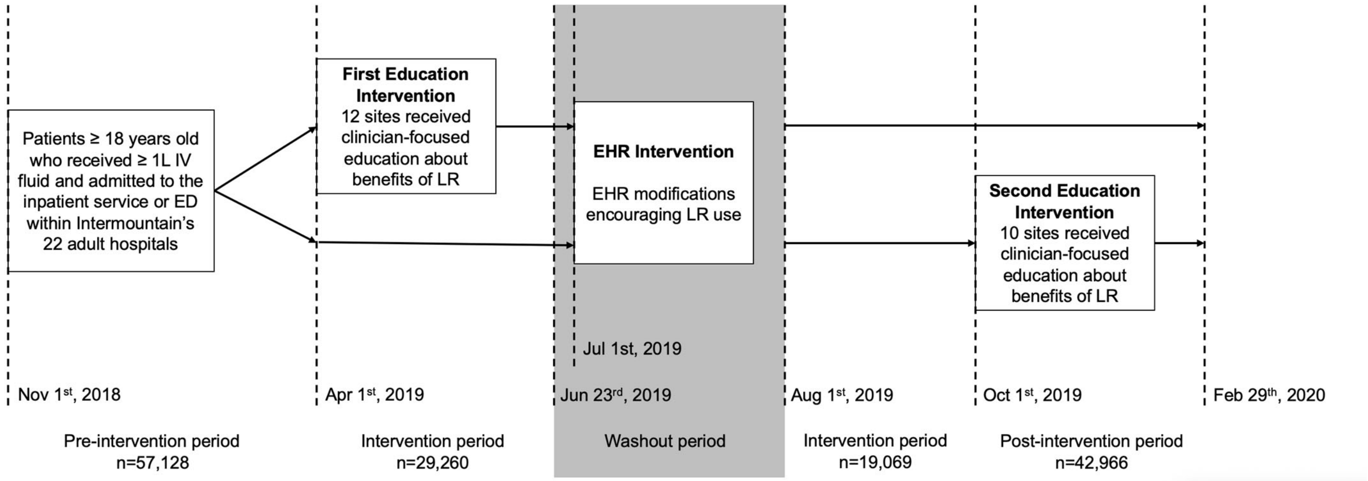

First Education Intervention: indicates education using standard communication channels (eg. Department meetings, email, huddle boards, etc.) for physicians, advanced practice clinicians, and pharmacists at the 12 hospitals designated in eTable1 as Phase 1. A mandatory computer-based training module was required of nurses in phase 1 and phase 2 hospitals during this Phase 1.
EHR intervention: Hospitals in Phase 1 and Phase 2 had EHR modifications turned on to encourage LR prescribing.
Second Education Intervention: Hospitals in Phase 2 received education using standard communication channels.

eFigure 3. Implementation Team Organizational Chart

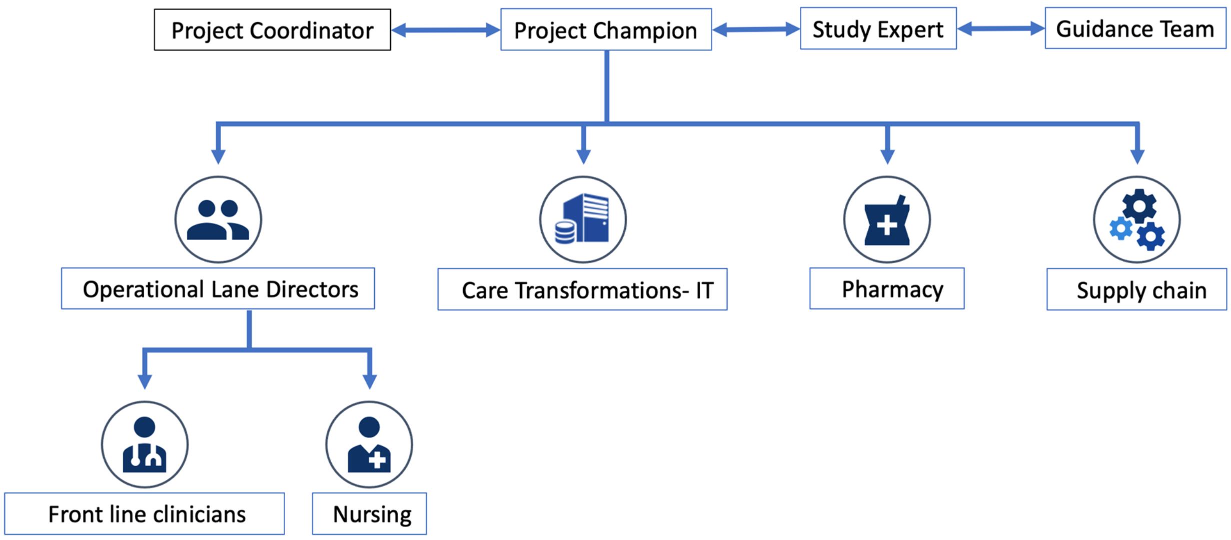

© 2022 Bledsoe J et al. *JAMA Network Open.*

eFigure 4. Example of Electronic Health Record Order Set

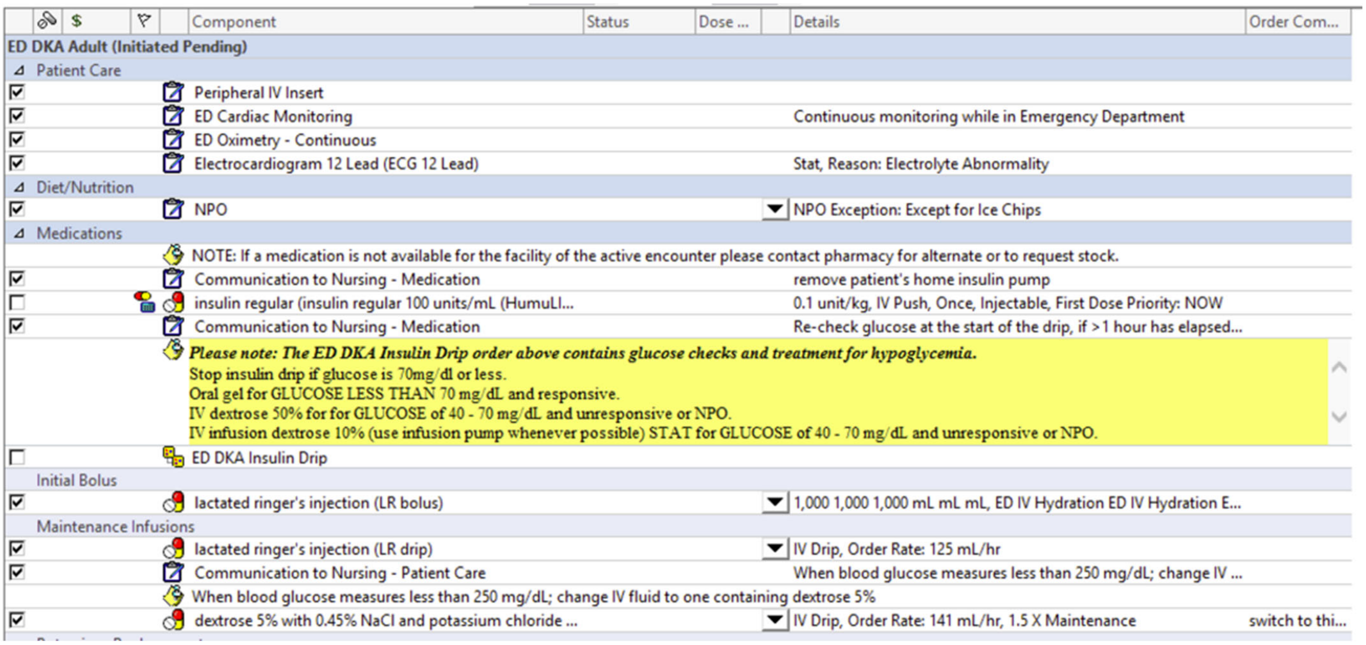

Lactated Ringer's fluids are pre-selected in this order set for emergency department patients with diabetic ketoacidosis as part of the implementation process. Normal saline is still an option, but ordering it requires first de-selecting lactated Ringer's and then selecting normal saline.

© 2022 Bledsoe J et al. *JAMA Network Open.*

eFigure 5. Best Practice Alert

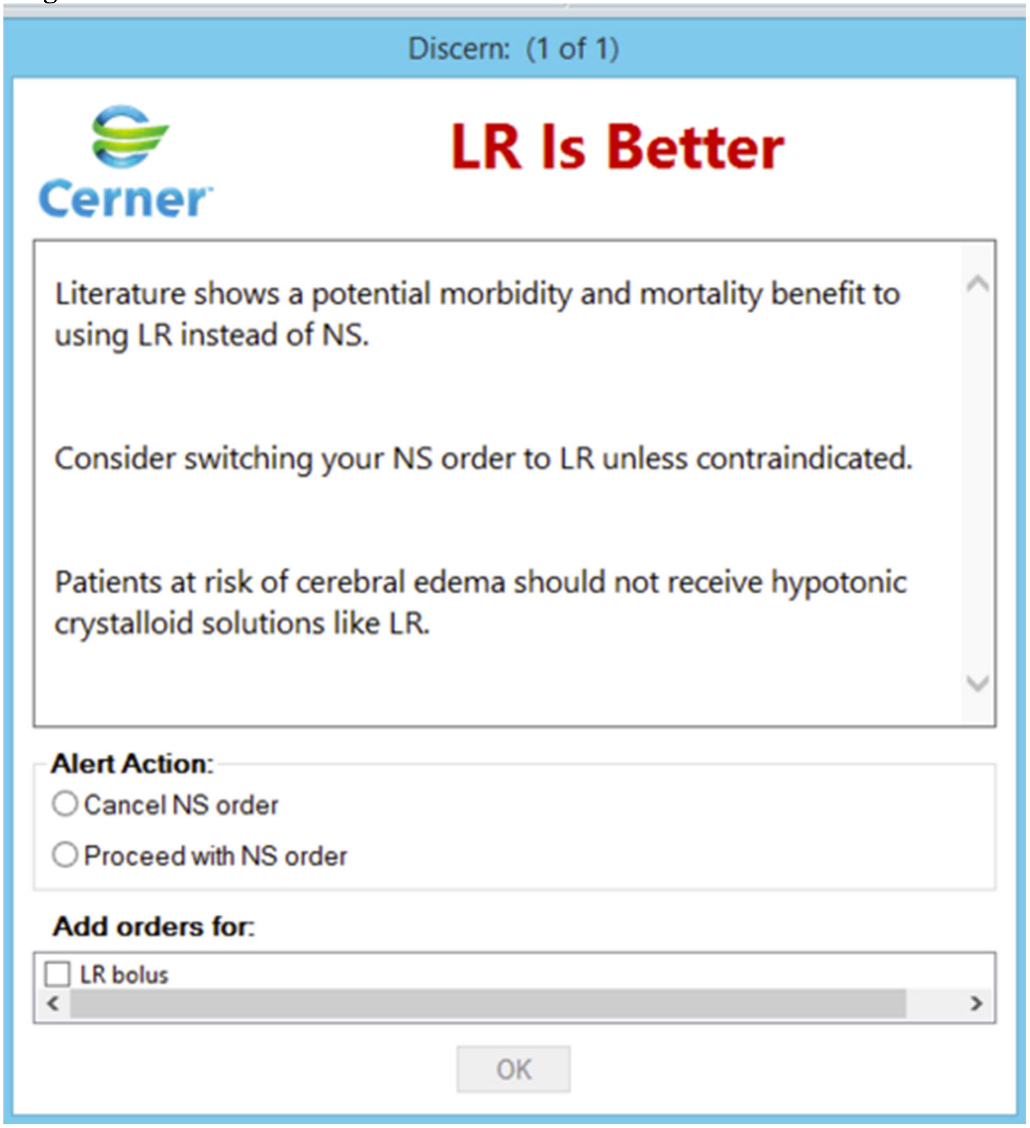

© 2022 Bledsoe J et al. *JAMA Network Open.*

eFigure 6. Sample Educational Materials Used for Implementation (Presentation)

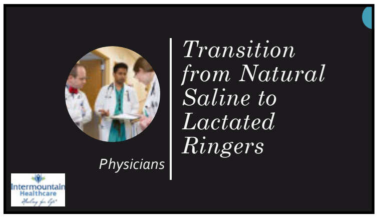

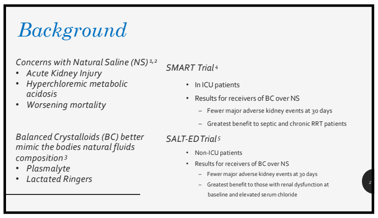

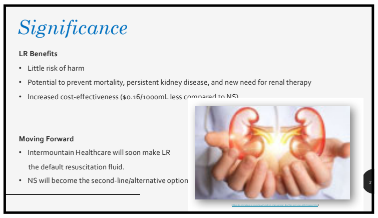

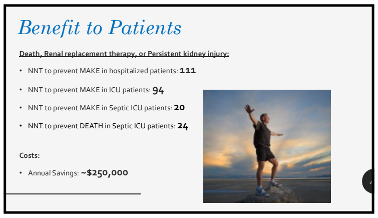

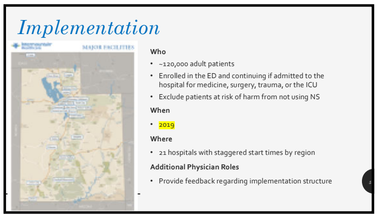

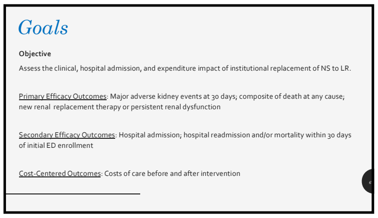

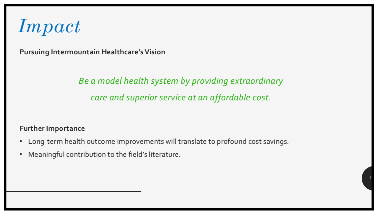

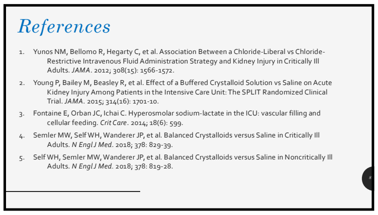

© 2022 Bledsoe J et al. *JAMA Network Open.*

eFigure 7. Sample Educational Materials Used for Implementation (Informational Flyer)

Quick-Fact Sheet for Lactated Ringer Transition – Physicians

Intermountain Healthcare
Healing for life

Background

While Normal Saline (NS) is currently the most commonly used intravenous fluid, recent literature has shown support for the use of Balance Crystalloids (BC) (including Lactated Ringer's and Plasmalyte) instead. Concerns continue to rise around NS causing acute kidney injury, hyperchloremic metabolic acidosis, and worsening of mortality.[1,2] BC are designed to closer mimic the body's natural electrolyte and solute compositions as shown in Table 1.

Table 1: Composition of Fluids[3]

Fluid Type	Na^+	K^+	Ca^{++}	Mg^{++}	Cl^-	Lactate	Osm	pH
Plasma	140	4	2.3	1	104	<1	285	7.4
Normal Saline	154				154		308	5
Lactated Ringer's (LR)	130	4	1.5		109	28	274	6.5
Plasmalyte	140	5		1.5	98		295	7.4

Two recent trials present convincing evidence for the use of BC over NS:

SMART Trial[4]: ICU patients using BC experienced significantly less major adverse kidney events at day 30 compared with those given NS; greatest benefit was in those with sepsis and chronic RRT

SALT-ED Trial[5]: Non-ICU patients using BC experienced significantly less major adverse kidney events at day 30 compared with those given NS; greatest benefit was in those with renal dysfunction at baseline and elevated serum chloride

Significance

Lactated Ringer's benefits:

- Little risk of harm
- Potential to prevent mortality, persistent kidney disease, and new need for renal therapy
- Increased cost-effectiveness at $0.16/1000mL less when compared to NS

Consequently, Intermountain Healthcare will alter system-wide guidelines and electronic order-sets to make LR the default resuscitation fluid with NS made as a second line/alternative option.

Implementation

Population: Inclusion: 118,000 adults >18; enrolled in the ED and continuing if admitted to the hospital for medicine, surgery, trauma, or the ICU

Exclusion: Patients with TBI, intra-cerebral hemorrhage, cerebral vascular accident, transient ischemic attack, hyponatremia, hypercalcemia, and other who may benefit from NS

Where: 21 hospitals with staggered start times by region based on resources, practice patterns of physicians, supply chain, and overall system efficiencies

When: 2019

Additional Physician Role: Provide feedback regarding implementation structure

Goals of Implementation

Objective: Assess the clinical, hospital admission, and expenditure impact of institutional replacement of NS to LR.

Primary Efficacy Outcomes: Major adverse kidney events at 30 days; composite of death at any cause; new renal replacement therapy or persistent renal dysfunction

Secondary Efficacy Outcomes: Hospital admission; hospital readmission and/or mortality within 30 days of initial ED enrollment

Cost-Centered Outcomes: Costs of care before and after intervention

Impact

This study aligns with Intermountain Healthcare's vision to provide the highest quality of care at the lowest possible cost. While immediate cost savings may not be profound, the real benefit will be synergistic with potential improvement to patient outcomes. Additionally, this study will enroll 10 times as many patients as previous studies, contributing meaningfully to the field's literature.

References

1. Yunos NM, Bellomo R, Hegarty C, et al. Association Between a Chloride-Liberal vs Chloride-Restrictive Intravenous Fluid Administration Strategy and Kidney Injury in Critically Ill Adults. *JAMA*. 2012; 308(15): 1566-1572.
2. Young P, Bailey M, Beasley R, et al. Effect of a Buffered Crystalloid Solution vs Saline on Acute Kidney Injury Among Patients in the Intensive Care Unit: The SPLIT Randomized Clinical Trial. *JAMA*. 2015; 314(16): 1701-10.
3. Fontaine E, Orban JC, Ichai C. Hyperosmolar sodium-lactate in the ICU: vascular filling and cellular feeding. *Crit Care*. 2014; 18(6): 599.
4. Semler MW, Self WH, Wanderer JP, et al. Balanced Crystalloids versus Saline in Critically Ill Adults. *N Engl J Med*. 2018; 378: 829-39.
5. Self WH, Semler MW, Wanderer JP, et al. Balanced Crystalloids versus Saline in Noncritically Ill Adults. *N Engl J Med*. 2018; 378: 819-28.

© 2022 Bledsoe J et al. *JAMA Network Open*.

eMethods.

There were 51 encounters with more than 24,000 mL received in 24 hours that were excluded due to implausible fluid volumes. Baseline serum creatinine values were obtained from up to one year prior to the date of admission for the index encounter. Baseline serum creatinine was calculated in cases where no value was available using the following formula:

$$Creatinine = 0.74 - 0.2\ (if\ female) + 0.08\ (if\ Black) + 0.003\ x\ age\ (in\ years)$$

Normal saline included fluids with 0.83-0.9% sodium chloride with or without dextrose or potassium chloride. Lactated Ringer's (LR) included LR with or without dextrose as well as an alternative proprietary balanced crystalloid solution (Plasma-Lyte). Bolus or maintenance infusions but not diluent or carrier fluids were included when calculating fluid administration volumes. The proportion of fluids received that were LR by volume (mL) was calculated with following formula:

$$Proportion\ BC = \frac{Lactated\ Ringer's\ volume + PlasmaLyte}{Normal\ Saline\ volume + Lactaed\ Ringer'svolume + PlasmaLyte}$$

Sepsis was identified per Sepsis-3 criteria[17] as the combination acute organ failure (Sequential Organ Failure Assessment score ≥2 points above pre-ED baseline) plus confirmed or suspected infection (based on collection of body fluid cultures and administration of an IV antimicrobial or oral vancomycin, fidaxomicin, or oseltamivir) prior to ED departure using an internally validated electronic data warehouse query.

Data analysis

We used a quasi-experimental analysis strategy, segmented linear regression, to support more robust causal inference from this non-randomized trial. For our effectiveness outcome, we first obtained the weekly adjusted MAKE30 (or other outcome) using binomial regression.

$$MAKE30 = \beta_Z \times (Age\ +\ Sex\ +\ Race/Ethnicity\ +\ Charlson\ +\ APS\ +\ Baseline\ Dialysis\ +\ Baseline\ Creatinine)$$

Variables in the model were *Age* (years); *Sex* (Male or Female); *Race/Ethinicity* (self-reported race/ethnicity, categorized as Hispanic/Latino, non-Hispanic American Indian/Alaska Native, non-Hispanic Asian, non-Hispanic Black, multiple races, non-Hispanic Native Hawaiian/Pacific Islander, non-Hispanic White, or unknown); *Charlson* comorbidity score (integers from 0 to 20); Acute physiology score (*APS Score,* integers from 0 to 52); *Baseline Dialysis* use (present/absent); and *Baseline Creatinine* (in mg/dL). β_Z represents the vector of coefficients for each variable. Risk adjusted models were then calibrated to predict MAKE30 at the same rate that was observed during the study. Formulas for weekly standardized MAKE30:

$$Adjusted\ MAKE30\ rate = \frac{Observed\ weekly\ rate}{Estimated\ weekly\ rate} \times\ Observed\ rate\ during\ study\ period$$

$$Observed\ weekly\ rate = \frac{Number\ of\ MAKE30\ outcomes\ among\ week's\ patients}{Number\ of\ patients\ for\ week}$$

$$Estimated\ weekly\ rate = \frac{Risk\ adjusted\ MAKE30\ outcomes\ among\ week's\ patients}{Number\ of\ patients\ for\ week}$$

$$Observed\ rate\ for\ study\ period = \frac{Number\ of\ MAKE30\ outcomes\ during\ study\ period}{Number\ of\ patients\ during\ study\ period}$$

© 2022 Bledsoe J et al. *JAMA Network Open.*

The Clopper-Pearson method was used to calculate 95% confidence intervals for the weekly risk-adjusted MAKE30 outcome. We then performed segmented linear regression based on fractional binomial regression to obtain interrupted time series estimates for the association between the intervention and the effectiveness outcomes according to the following formula.

$$\frac{Number\ of\ patients \times\ Adjusted\ MAKE30\ rate}{Number\ of\ patients} = \beta_0 + \beta_{pre}(time) + \beta_{step}(Post\ Implementation) + \beta_{post}(timeAfter)$$

In this formula, *time* is the integer count of weeks during study period from 1 to 69; *Post Implementation* is binary where 0 represents pre-implementation and 1 represents post implementation; *timeAfter* is the integer count of weeks during the post-implementation phase of the study from 0 for weeks pre-implementation and 1 to 33 weeks post-implementation; and *rate of Adjusted MAKE30* and *# patients* are week-specific rates and counts, respectively, from the cohort for a total of 69 datapoints. The week-on-week trend for the outcome pre- and post-intervention is obtained from coefficients β_{pre} and β_{post}, respectively, while the step-off effect is provided by the coefficient β_{step}. Absolute and relative risk difference was calculated with regard to the final week of the study per the following formulas:

$$Absolute\ risk\ difference = Estimated\ MAKE30\ with\ intevention - Estimated\ MAKE30\ without\ intervention$$

$$Relative\ risk\ difference = \frac{Estimated\ MAKE30\ with\ intevention - Estimated\ MAKE30\ without\ intervention}{Estimated\ MAKE30\ without\ intervention}$$

We used a similar segmented linear regression approach based on beta regression with a logit link to obtain interrupted times series estimates of the association between implementation interventions and the proportion of LR received:

$$Proportion\ LR = \beta_0 + \beta_{pre1}(time) + \beta_{pre2}(timeEducation1) + \beta_{step}(Post\ Implementation) + \beta_{post}(timeEducation2)$$

In this formula, *time* and *Post Implementation* are the same as the MAKE30 model above, and *time Education1* and *time Education2* is the integer count of weeks after the educational interventions from 0 during pre-education time frames and 1 to 47 for *time Education1* and 1 to 21 for *time Education2.*
The binary variable, *Post Implementation*, allows for analyzing and immediate change, or step-off, of the intervention. The time variables that count whole weeks from a specific time point (e.g., *time, timeAfter, time Education1, time Education2*) permit an analysis of trend, or slope during the period where the time count is greater than zero. Coefficients provide trend and step-off effect estimates analogous to the prior equation.

© 2022 Bledsoe J et al. *JAMA Network Open.*

eTable 3. ICD-10 Root Codes Occurring in 5% or More of the Study Population as the Primary (First) Discharge Diagnosis

Truncated ICD10	ICD10 Description	n (%)
R10	Abdominal and pelvic pain	12230 (8.2%)
O80	Encounter for full-term uncomplicated delivery	5728 (3.9%)
R11	Nausea and vomiting	4276 (2.9%)
N20	Calculus of kidney and ureter	4142 (2.8%)
R51	Headache	3263 (2.2%)
Z98	Other postprocedural states	2739 (1.8%)
R07	Pain in throat and chest	2722 (1.8%)
N39	Other disorders of urinary system	2531 (1.7%)
J18	Pneumonia, unspecified organism	2524 (1.7%)
K52	Other and unsp noninfective gastroenteritis and colitis	2380 (1.6%)
R55	Syncope and collapse	2320 (1.6%)
Z96	Presence of other functional implants	2199 (1.5%)
A41	Other sepsis	2178 (1.5%)
R42	Dizziness and giddiness	1932 (1.3%)
F10	Alcohol related disorders	1849 (1.2%)
M17	Osteoarthritis of knee	1773 (1.2%)
G43	Migraine	1692 (1.1%)
J96	Respiratory failure, not elsewhere classified	1649 (1.1%)
J10	Influenza due to other identified influenza virus	1403 (0.9%)
E11	Type 2 diabetes mellitus	1314 (0.9%)
S72	Fracture of femur	1280 (0.9%)
K80	Cholelithiasis	1257 (0.8%)
K92	Other diseases of digestive system	1239 (0.8%)
I63	Cerebral infarction	1231 (0.8%)
N12	Tubulo-interstitial nephritis, not spcf as acute or chronic	1229 (0.8%)
K56	Paralytic ileus and intestinal obstruction without hernia	1225 (0.8%)
K57	Diverticular disease of intestine	1220 (0.8%)
I48	Atrial fibrillation and flutter	1160 (0.8%)
M54	Dorsalgia	1150 (0.8%)
R19	Oth symptoms and signs involving the dgstv sys and abdomen	1127 (0.8%)
R53	Malaise and fatigue	1105 (0.7%)
L03	Cellulitis and acute lymphangitis	1083 (0.7%)
M16	Osteoarthritis of hip	1061 (0.7%)
R65	Symp and signs specifically assoc w sys inflam and infct	1047 (0.7%)
R50	Fever of other and unknown origin	970 (0.7%)
E87	Other disorders of fluid, electrolyte and acid-base balance	963 (0.6%)
K85	Acute pancreatitis	958 (0.6%)
Z34	Encounter for supervision of normal pregnancy	957 (0.6%)
I21	Acute myocardial infarction	949 (0.6%)
Z3A	Weeks of gestation	943 (0.6%)
E86	Volume depletion	909 (0.6%)
B34	Viral infection of unspecified site	899 (0.6%)
G93	Other disorders of brain	859 (0.6%)
R00	Abnormalities of heart beat	855 (0.6%)

© 2022 Bledsoe J et al. *JAMA Network Open.*

O82	Encounter for cesarean delivery without indication	836 (0.6%)
N17	Acute kidney failure	812 (0.5%)
E66	Overweight and obesity	808 (0.5%)
N83	Noninflammatory disord of ovary, fallop and broad ligament	802 (0.5%)
I10	Essential (primary) hypertension	763 (0.5%)
R56	Convulsions, not elsewhere classified	755 (0.5%)
F41	Other anxiety disorders	712 (0.5%)

eFigure 8. Forest Plot of Relative Difference in Incidence Rate of MAKE30 for Subgroups

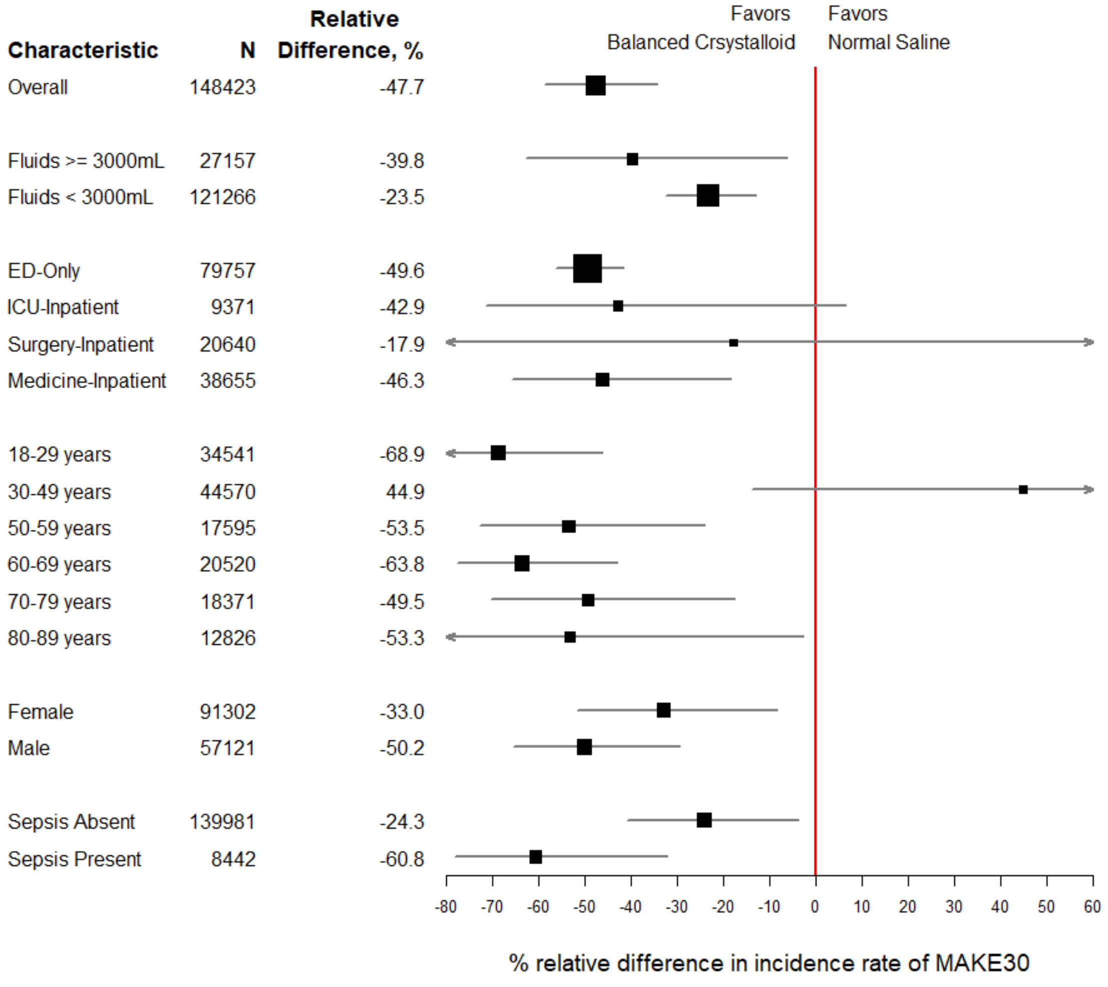

© 2022 Bledsoe J et al. *JAMA Network Open.*

eFigure 9. Observed and Risk-Adjusted Rate of MAKE30 in Patient Subgroups

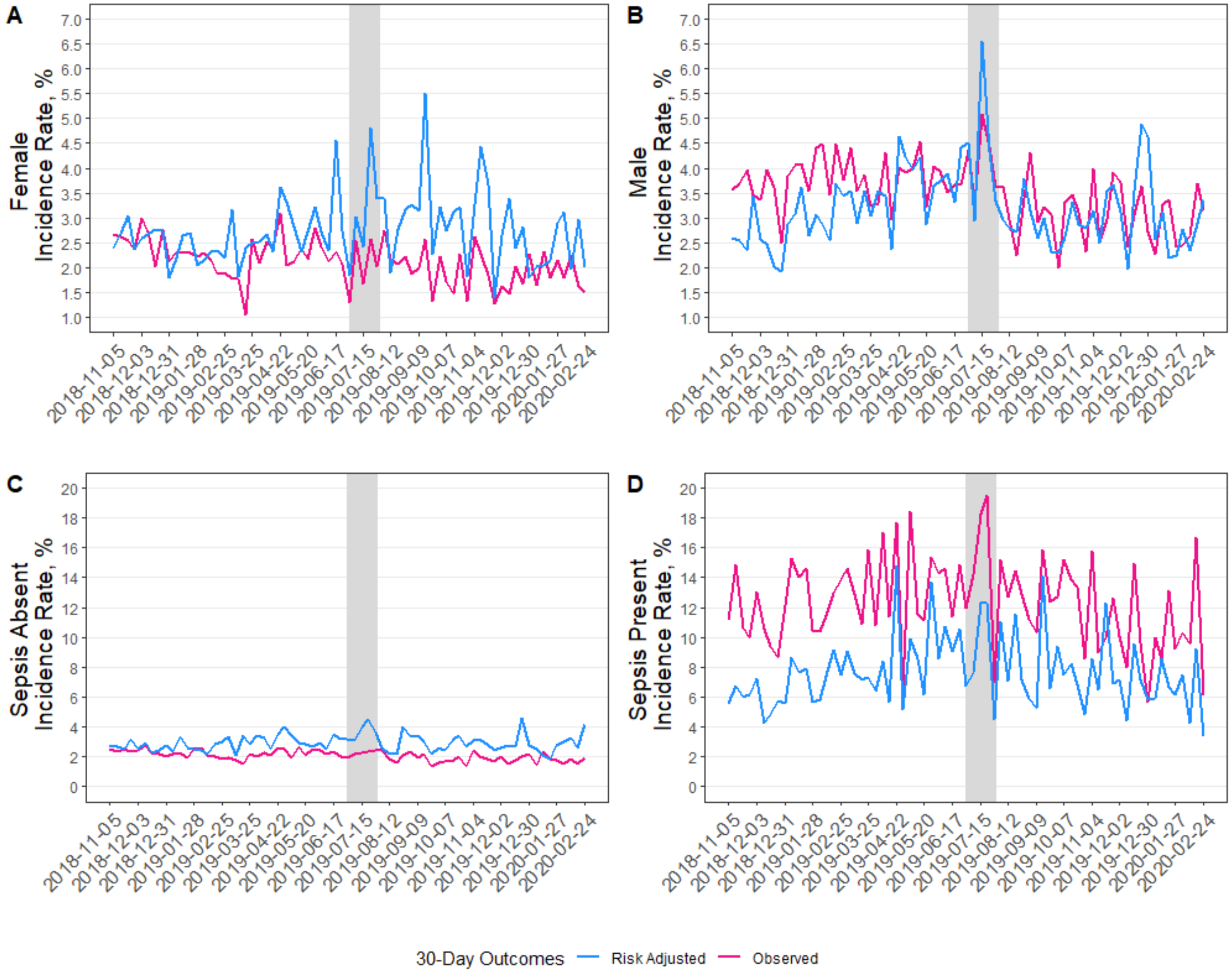

Figures depict major adverse kidney outcomes at 30 days (MAKE30, a composite of persistent renal dysfunction, new renal replacement therapy, and mortality at 30 days) among patients admitted in the indicated week for patient who are A) female or B) male or who have C) sepsis absent or D) sepsis present during their admission.

© 2022 Bledsoe J et al. *JAMA Network Open.*

eFigure 10. Observed and Risk-Adjusted Incidence Rate of MAKE30 by Patient Admission Type

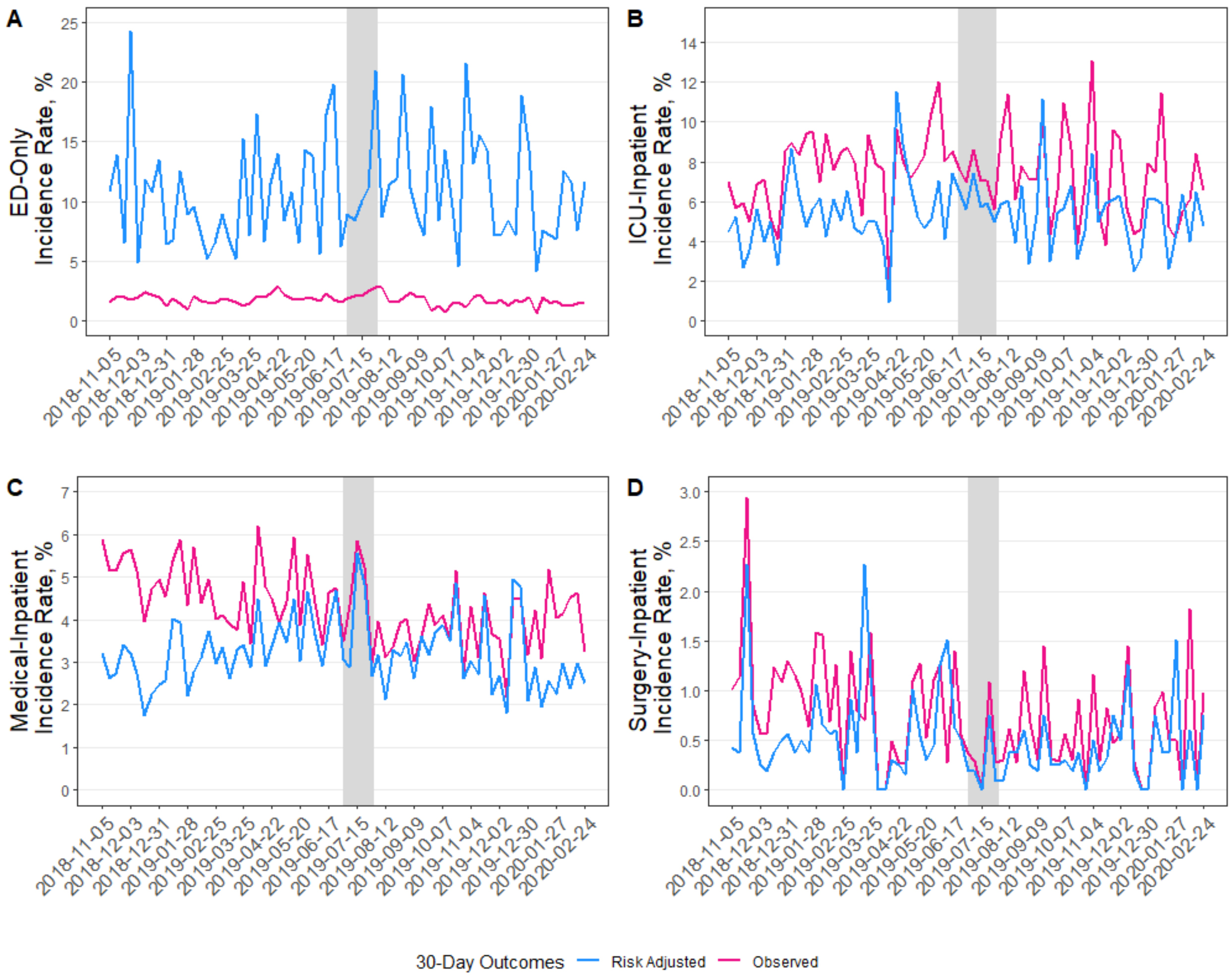

Figures depict major adverse kidney outcomes at 30 days (MAKE30, a composite of persistent renal dysfunction, new renal replacement therapy, and mortality at 30 days) among patients admitted in the indicated whose highest level of care was A) intensive care unit (ICU), B) emergency department (ED), C) inpatient surgery, and D) inpatient.

© 2022 Bledsoe J et al. *JAMA Network Open.*

eFigure 11. Observed and Risk-Adjusted Incidence Rate of MAKE30 in Patient Subgroups by Age

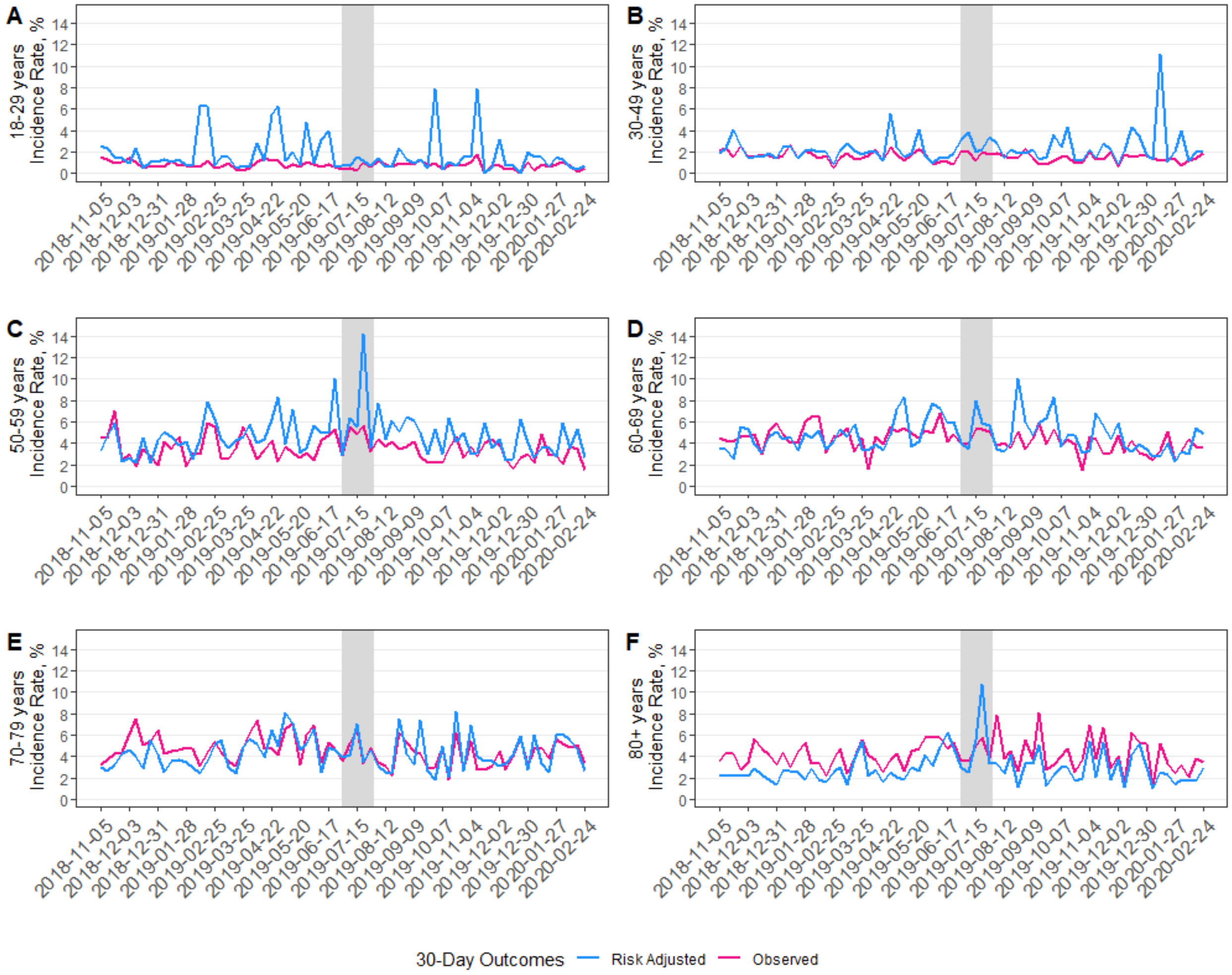

Figures depict major adverse kidney outcomes at 30 days (MAKE30, a composite of persistent renal dysfunction, new renal replacement therapy, and mortality at 30 days) among patients admitted in the indicated week for patient age subgroups: A) 18 to 29 years, B) 30 to 49 years, C) 50 to 59 years, D) 60 to 69 years, E) 70 to 79 years, and F) ≥80 years.

© 2022 Bledsoe J et al. *JAMA Network Open.*
